# Application of a Novel Instantized Glycerol Monooleate Ingredient in a Protein-Stabilized Oil-In-Water Emulsion †

**DOI:** 10.3390/foods9091237

**Published:** 2020-09-04

**Authors:** Chia Chun Loi, Graham T. Eyres, Patrick Silcock, E. John Birch

**Affiliations:** Department of Food Science, University of Otago, P.O. Box 56, Dunedin 9054, New Zealand; marcus.loi@postgrad.otago.ac.nz (C.C.L.); pat.silcock@otago.ac.nz (P.S.); john.birch@otago.ac.nz (E.J.B.)

**Keywords:** glycerol monooleate, sodium caseinate, whey protein concentrate, creaming stability, oxidative stability, volatile analysis

## Abstract

Glycerol monooleate (GMO), casein and whey proteins are surfactants that can stabilize emulsion systems. This study investigates the impact of instantized GMO powders on creaming stability and oxidative stability in protein-stabilized emulsions. Model emulsions with bulk GMO, two instantized GMO powders, and two controls (without GMO) were produced by microfluidization. The droplet size, ζ-potential, viscosity, and creaming index of the emulsions were measured, while oxidative stability was evaluated by analysis of volatile compounds during storage (28 days, 45 °C) using gas chromatography mass spectrometry. Emulsions with GMO produced smaller average droplet sizes (180.0 nm) with a narrower distribution (polydispersity index of 0.161) compared to the controls (197.6 nm, 0.194). The emulsion stability of instantized emulsions was as good as bulk GMO, which were both better than controls. Based on the relative abundance of 3-octen-2-one, 2,4-heptadienal isomer 2, and 3,5-octadien-2-one isomer 1, the oxidative stability of the instantized emulsions was not significantly different from controls; however, bulk GMO emulsion showed significantly lower stability than controls. Instantized GMO powders can successfully produce physically stable protein-stabilized emulsions with good oxidative stability in a convenient powdered format.

## 1. Introduction

Protein-stabilized emulsions are examples of oil-in-water emulsions that have been used to deliver nutrients, bioactive compounds, and flavors [1]. The most common food emulsions are yogurt, cream, coffee creamer, milk, and plant-based beverages [2]. Proteins are the main group of emulsifiers that stabilize oil droplets in the aqueous phase. Many proteins are amphiphilic molecules containing both hydrophilic and hydrophobic structures, which make them good emulsifiers. The amphiphilic nature of protein allows them to adsorb at the oil-water interface and form a protective film surrounding the oil droplets [3]. The adsorbed proteins at the interface provide repulsive forces, such as steric and electrostatic forces, to stabilize the oil droplets in the aqueous phase [4]. However, oil droplets can simultaneously undergo multiple physical destabilization mechanisms such as creaming, flocculation, and coalescence that eventually lead to complete separation of oil and aqueous layers.

Casein and whey proteins are milk proteins primarily used in food formulations due to their outstanding emulsifying properties [5]. Milk proteins can also adsorb to the air-water interface under agitation leading to foam formation. Foaming in milk beverages is undesirable as it can lead to inconsistent product quality, higher product loss, and lower productivity. Low-molecular-weight emulsifiers, such as monoglycerides, can be added to the emulsion to increase emulsion stability [6] and reduce foam formation during homogenization [7].

Creaming is the most common destabilization mechanism that occurs in oil-in-water emulsions such as milk beverages [2]. Creaming is an upward movement of dispersed oil droplets in the emulsion due to the density difference between oil and aqueous phases upon standing. The cream layer is usually easy to re-suspend into the emulsion upon agitation, as the oil droplets retain their integrity due to the absence of physical and chemical interactions. Previous work [8] investigated various monoglyceride compositions in model emulsions, and the results showed that glycerol monooleate (GMO) produced smaller oil droplets with narrow size distribution and greater stability towards creaming during storage when compared to the control (no GMO).

GMO is an unsaturated monoglyceride with only one of the hydroxyl groups of glycerol esterified with an unsaturated fatty acid, i.e., oleic acid. The oil-soluble nature of GMO means that it has high solubility in oil and poor dispersibility in water and is thus not suitable for many food formulations. However, if GMO could be transformed into an instantized powder with good dispersibility in water using spray-drying it could produce a suitable food ingredient. Spray-drying is the most commonly available technique in the food industry to transform hydrophobic materials, such as oil and bioactive compounds, into instantized powders with good water dispersibility and protection against lipid oxidation [9]. The unsaturated fatty acids in GMO are prone to oxidation, which is the root cause of rancidity and off-flavor in these ingredients. Measurement of volatile secondary oxidation products by headspace solid-phase microextraction (HS-SPME) with gas chromatography-mass spectrometry (GC-MS) has been used extensively to study the progression of lipid oxidation [10].

In our previous work [11], we reported on the feasibility of using spray-drying to prepare a novel instantized emulsifier based on GMO that can be easily reconstituted in water and on the properties of the powders. From this work, the two instantized GMO powders that exhibited the lowest surface oil (3%), good dispersibility in water (74–87%), and the smallest change in droplet size after reconstitution were selected for the current study. The current study aims to evaluate the effect of instantized GMO powder on the physicochemical properties, creaming stability, and oxidative stability in protein-stabilized emulsions. The key comparisons were between emulsions with bulk GMO (bGMO) and instantized GMO powder, and between emulsions with GMO and control emulsions without GMO.

## 2. Materials and Methods

### 2.1. Materials

Glycerol monooleate 90% (Radiamuls MG 2905K) was provided by Oleon (Klang, Malaysia). Whey protein concentrate 80% (WPC) and sodium caseinate were sourced from Tatua Co-operative Dairy Company Ltd. (Morrinsville, New Zealand). Maltodextrin powders were sourced from Hawkins Watts Ltd. (Auckland, New Zealand) (dextrose equivalent (DE) 10) and Davis Food Ingredients (Auckland, New Zealand) (DE 18), respectively. Canola oil (refined, bleached, and deodorized) and sugar were purchased from retail stores and sodium azide was obtained from Sigma-Aldrich (St. Louis, MO, USA). Hexane (95%, Unilab laboratory reagent) and methanol (99.8%, Univar analytical reagent) were sourced from Ajax Finechem (Auckland, New Zealand), and chloroform (EMSURE grade) from Merck (Darmstadt, Germany).

### 2.2. Preparation of Instantized GMO Powders by Spray-Drying

Instantized GMO powders (GMO + DE10 and GMO + DE18) were prepared as described in our previous work [11]. In brief, microfluidization was used to produce emulsions as per the formulation shown in Table 1a at constant total solids of 40% *w/w* and then dried using a laboratory scale spray dryer (LabPlant SD-05 model; Keison Products, Essex, England) on the same day each emulsion was prepared. Sodium stearate was added as a component of GMO at 4% *w/w* to form a suitable emulsion for drying. All water-phase ingredients were first reconstituted in deionized water at 50 °C using an Ultra-Turrax (IKA-Werke GmbH and Co. KG, Stufen, Germany) at 13,000 rpm and was simultaneously heated to 75 °C. GMO and canola oil was then added to the water-phase and homogenized for 15 min at 75 °C to form a coarse emulsion. Fine emulsions were produced using a Microfluidizer^®^ (Microfluidics International Corporation, Westwood, MA, USA) at 55 MPa and 65 °C using a single cycle. The fine emulsion was dried using the spray-dryer with an infeed temperature of 55 °C with a 1.0 mm diameter atomizer nozzle.

Powder properties of the instantized GMO powders are shown in Table 1b. Moisture content, dispersibility, surface oil, and total oil of the powders were determined as described in our previous work [11]. Moisture content was determined by loss on drying at 103 ± 2 °C and was calculated using Equation (1):

(1) $$\mathrm{Moisture}=\frac{\mathrm{Initial}\;\mathrm{powder}\;\mathrm{weight}-\mathrm{Dried}\;\mathrm{powder}\;\mathrm{weight}}{\mathrm{Initial}\;\mathrm{powder}\;\mathrm{weight}}\times 100$$

Dispersibility of the powders was measured by reconstituting the powder in water and evaluating the amount of powder that can pass a 250 μm test sieve after 20 complete stirring movements according to the GEA Niro Method No. A 6a [12] with some modifications. The dispersibility of the powder was calculated using Equation (2):

(2) $$\mathrm{Dispersibility}=\frac{\left(a+b\right)\times d}{b\times \frac{100-c}{100}}$$

where

a = weight of water added to the powder in g;
b = weight of powder used in g;
c = moisture content (%) in the powder;
d = dry matter (%) in the reconstituted emulsion after it has passed through the sieve.

Encapsulation efficiency of the instantized GMO powders is a measure of the amount of oil-soluble ingredients encapsulated by matrix, determined using Equation (3):

(3) $$\mathrm{Encapsulation}\;\mathrm{efficiency}=\frac{\mathrm{Total}\;\mathrm{oil}-\mathrm{Surface}\;\mathrm{oil}}{\mathrm{Total}\;\mathrm{oil}}\times 100$$

Surface oil was determined by washing the powders several times with hexane according to González et al. [13] with some modifications. Total oil content was determined by oil extraction using methanol and chloroform according to the procedure described by Sarkar et al. [14] with some modifications.

### 2.3. Model Emulsions

Five model emulsions with different emulsifier systems (Table 2) were produced by microfluidization according to the procedure described in our previous work [8]. All samples were prepared in duplicate and in a randomized block design on the same day. Water-phase ingredients consisted of sodium caseinate, whey protein concentrate, caster sugar, and sodium azide, reconstituted in deionized water at 50 °C. Instantized GMO powder or maltodextrin was only added to the water-phase when required in the formulation. The level of sugar, sodium caseinate, and whey protein concentrate in the samples were intended to match typical commercial dairy beverages. Sodium azide was employed to prevent the proliferation of microorganisms during storage. The water-phase was heated to 75 °C and continuously agitated using an Ultra-Turrax at 10,000 rpm. GMO, when required, was dissolved in the canola oil at 60 °C, which was then homogenized with the water-phase to form a coarse emulsion. The visual appearance of the foaming behavior after formation of the coarse emulsion was recorded by using the camera (8 MP) of an iPhone 5S (Apple Inc., Cupertino, CA, USA). Fine emulsions were prepared using a Microfluidizer^®^ at 55 MPa and 65 °C with a single cycle. The Microfluidizer^®^ was pre-heated by circulating water at 70 °C for 2 min.

Model emulsions were filled into either clear test tubes (16 mm od × 150 mm height), 100 mL bottles, or 20 mL headspace vials with Teflon-lined septa for measurement of the creaming index, emulsion stability, and oxidative stability, respectively. Emulsion samples were evaluated for their physical and oxidative stability under accelerated conditions at 45 °C for 28 days, as the maximum cream layer was obtained in the emulsion at 28 days and very little change to the cream layer was observed after this period.

### 2.4. Characterization of Model Emulsions

#### 2.4.1. Emulsion Particle Size and Polydispersity

Emulsion droplet sizes and size range distributions were determined using dynamic light scattering using a Malvern Zetasizer Nano S (Malvern Instruments Ltd., Malvern, Worcestershire, UK). Measurements were conducted as described by Loi, Eyres, and Birch [8]. The assumption of the sphericity of the emulsion droplets was verified using an optical microscope. Samples were diluted with a 6% sucrose solution (1/1000) and then analyzed for 60 s at 25 °C in duplicate. The average droplet size was calculated as the intensity-weighted mean diameter (Z-average), and the polydispersity index was used to measure the degree of non-uniformity of the size distribution.

#### 2.4.2. ζ-Potential

The surface charge or ζ-potential of the emulsion samples was evaluated using a Malvern Zetasizer Nano S, as previously reported [8]. Emulsion samples were diluted with a 6% sucrose solution (1/20) for analysis and each measured in duplicate.

#### 2.4.3. Viscosity

Viscosity of the emulsions was measured with a Haake Rheostress 1 rheometer (Karlsruhe, Germany) using a double gap geometry (rotor DG43 DIN53544 Ti; measuring cup DG42 RO) at 20 °C, as previously described [15]. Each sample was measured in duplicate over a shear rate range of 1 to 500 s^−1^.

#### 2.4.4. pH

The pH of emulsions was tested at room temperature using a pH meter (pH209, HANNA Instruments, Woonsocket, RI, USA).

#### 2.4.5. Creaming Index

The creaming index of the emulsions was obtained by a visual observation method as described previously [8]. The thickness (mm) of the cream layer were measured on emulsions stored in sealed clear test tubes and creaming index was calculated according to McClements [16].

#### 2.4.6. Emulsion Stability

All emulsions for the stability test were sealed in 100 mL bottles, and the bottles were agitated until no visual cream separation before conducting any analysis at each storage time. Samples were evaluated in terms of droplet size, polydispersity index, ζ-potential, viscosity, and pH.

#### 2.4.7. Volatile Analysis Using Headspace Solid-Phase Microextraction (HS-SPME) and GC-MS

The extraction of volatiles in the samples was conducted as per Loi, Eyres, and Birch [15]. Emulsions (5 g) were stored in sealed 20 mL headspace vials at 45 °C for 1–28 days to evaluate changes in volatile compounds. Samples were removed at specified time intervals and stored at −20 °C prior to volatile analysis.

The change in headspace volatiles during storage was analyzed using an Agilent GC 6890N gas chromatograph (Agilent Technologies, Palo Alto, CA, USA) attached to an Agilent 5975B VL MSD quadrupole mass spectrometer (Agilent Technologies, Wilmington, DE, USA), as described previously [15] with some modifications. Vials were incubated at 45 °C for 5 min, then the sample was extracted using SPME (50/30 μm divinylbenzene-carboxen-polydimethylsiloxane; Supelco, Bellefonte, PA, USA) for 40 min. Six analyses were performed on each emulsion formulation (two emulsions × three analytical replicates per batch).

After extraction, the SPME fiber was desorbed at 240 °C for 5 min (2 min splitless, 3 min split) using helium gas. The chromatographic separation was performed on a 60 m × 0.32 mm id × 0.5 μm Zebron ZB-WAX column (Phenomenex, Torrance, CA, USA) with helium as the carrier gas (1 mL min^−1^). Mass spectra (29–300 m/z) were collected at a 5.1 scan s^−1^ with the quadrupole set to 150 °C with an ionization energy of 70 eV. The carryover was prevented between GC runs by conditioning the fiber for 2 min at 270 °C, 50 mL min^−1^. The lack of carryover was confirmed using an empty vial blank and a deionized water blank at the start, middle, and end of a randomized block of 15 samples run on each day. All analyses were conducted over six consecutive days.

GC-MS data was processed using Agilent Enhanced MSD Chemstation software (version F.01.01.2317, Agilent Technologies Inc., Santa Clara, CA, USA). Peak areas of the volatile compounds were expressed using the total ion count (TIC) chromatogram. Volatile compounds were identified based on their mass spectra compared to the National Institute of Standards and Technology database (NIST14). Identifications were supported by comparing retention indices (RI) to literature databases. Linear retention indices (RI) were calculated using a C9–C17 n-alkane series.

Calibration curves for hexanal, octanal, and 1-octen-3-ol were prepared using a standard addition method and the linear region in the range of 0–900 µg/L was used for semi-quantification. All samples were measured with six replicates (*n* = 6).

#### 2.4.8. Statistical Analysis

Statistical comparison of the results was conducted using IBM SPSS Statistics version 23 (IBM Corporation, Armonk, New York, NY, USA). One-way analysis of variance (ANOVA) was carried out to determine significant differences between the results, followed by Tukey’s post hoc test for pairwise comparison. All tests were performed at the 95% confidence level. Principal component analysis (PCA) was carried out on the peak areas of all volatile compounds that increased significantly over time using The Unscrambler X version 10.5 (CAMO, Oslo, Norway) and all variables in the PCA were standardized (1/standard deviation).

## 3. Results and Discussion

### 3.1. Observation of Foaming Behavior during Homogenization

**Figure 1:**
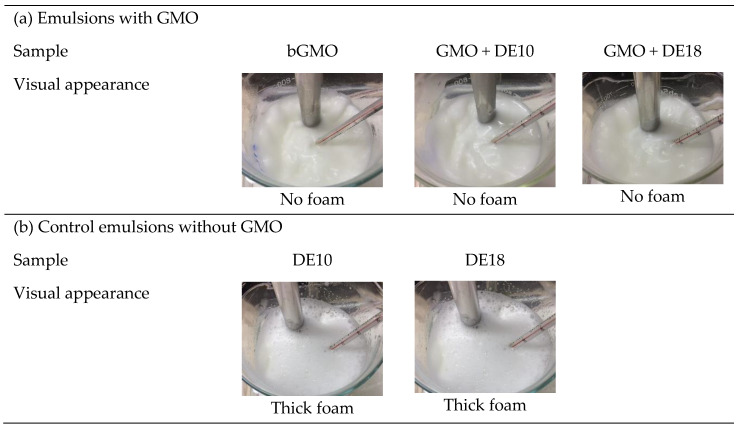
Visual appearance of model emulsions (**a**) with and (**b**) without GMO during high-shear homogenization.

Figure 1 shows the visual appearance of the model emulsions formulated with different emulsifier systems during high-shear homogenization. The foams in the emulsions disappeared after addition of GMO (bGMO, GMO + DE10 and GMO + DE18) during homogenization, but a substantial amount of foam remained in the control emulsions (DE10 and DE18). According to Hanselmann and Windhab [17], the agitation between protein and sugar in the solution leads to foam formation. In addition to emulsification, GMO also exhibits anti-foaming properties in protein-stabilized emulsions that could increase productivity and product yield through foam reduction.

### 3.2. Droplet Size and Polydispersity Index

Table 3 shows the droplet size and polydispersity index of model emulsions formulated with different emulsifier systems over 28 days of storage at 45 °C. The average droplet size of the control emulsions (DE10 and DE18; without GMO) differed significantly from emulsions containing GMO (bGMO, GMO + DE10 and GMO + DE18) (*p* < 0.05). Fresh and aged emulsions with GMO had significantly smaller droplet sizes (172–186 nm) than the controls without GMO (194–200 nm). This observation agrees with previous findings [8] that emulsions with monoglycerides form smaller droplets than controls. However, emulsion droplet sizes were the same (*p* > 0.05) for bulk GMO and instantized GMO powders of different DE values of maltodextrin. The sodium stearate from the GMO powders was very low (around 80 ppm in the emulsions) and did not affect the droplet size. Both controls with either maltodextrin DE10 and DE18 also had the same droplet sizes, illustrating no apparent effect of maltodextrin type on the emulsion properties.

Droplet sizes for all emulsion samples were stable during 28 days of storage at 45 °C. This finding concurs with previous works on protein-stabilized emulsions that indicated droplet sizes were stable during storage, without any significant droplet growth due to flocculation, coalescence, and Ostwald ripening [8,18,19,20].

**Figure 2:**
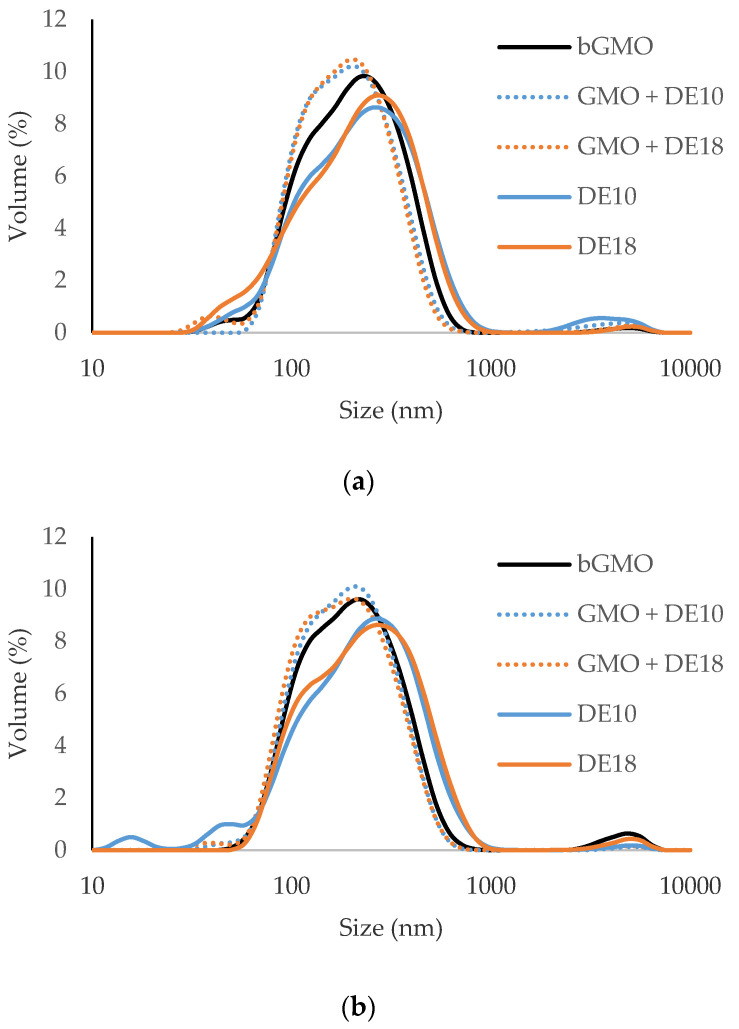
Volume-weighted size distribution of (**a**) fresh and (**b**) 28 days-aged model emulsions.

Table 3b shows the polydispersity index of emulsions over 28 days of storage at 45 °C. All emulsions with GMO and controls had a polydispersity index of 0.2 or lower, which indicated narrow size-range distributions. The volume-weighted droplet size distribution showed that all fresh and 28-day aged emulsions had a pseudo-monomodal size distribution (Figure 2), supporting the polydispersity index results. The fine droplets around 20 nm in the 28-day aged DE10 sample were less than 1.5%, hence did not affect its polydispersity index. The polydispersity index values were 18% lower for emulsions formulated with GMO compared to the controls. Regardless of storage time, emulsions with bulk GMO and instantized GMO had smaller average droplet sizes and narrower droplet size distributions than controls. Both control emulsions with maltodextrin with different DE values did not show any significant difference in terms of droplet size and polydispersity index from each other. This observation reaffirmed that maltodextrin at this level had no significant effect on the droplet size and its size-range distribution, which is in line with the findings by Gharsallaoui et al. [21]. In our previous study, the emulsions with a sodium stearate concentration up to 120 ppm did not show any significant change to the emulsion droplet size when compared to the control emulsions without sodium stearate [8]. All emulsions had similar protein composition and concentration, thus suggesting that GMO is responsible for the reduction in droplet size of the emulsions.

### 3.3. ζ-Potential

Table 4 shows the ζ-potential of model emulsions formulated with different emulsifier systems over 28 days of storage at 45 °C. All fresh emulsions (Day 0) formulated with different emulsifier systems had a ζ-potential in the range of −51 to −53 mV. In order to simplify the discussion, as all emulsions in this study exhibited negative ζ-potential values, the negative sign will be ignored and only the magnitude will be discussed. Hence, an increase in ζ-potential means the increase in the negativity of the ζ-potential. The ζ-potential provides information on the repulsive forces at the oil-water interface, which can explain droplet stability in the emulsions [22]. All emulsions with different emulsifier systems showed statistically significant differences after Day 7 (*p* < 0.05) when comparing the samples at each timepoint, but the small differences in ζ-potential (<3 mV) are not useful to distinguish the stability of emulsifier systems, and all samples can be considered stable [16]. Sodium stearate in the instantized powders increased the ζ-potential of the emulsions, similar to previous findings [8]. The increase in ζ-potential by sodium stearate was potentially due to the adsorption of anionic molecules at the oil-water interface, thereby increasing the ionic repulsive force among the oil droplets. The similarity in ζ-potential of the different samples at the same storage time indicates that the emulsion droplets are predominantly stabilized by protein [23] and not much protein displacement by the emulsifier is taking place. It is hypothesized that sodium caseinate contributes to the negative ζ-potential in the soluble form and the slightly lower ζ-potential value after storage might be due to partial precipitation of sodium caseinate [24]. All emulsions showed a decreasing trend in ζ-potential with storage time (*p* < 0.05), with a small reduction of ζ-potential (<5 mV) over 28 days of storage. However, this may not have any practical implication because the oil droplets still retained high repulsive forces (−47 to −50 mV). Ross and Morrison [25] stated that oil droplets with a ζ-potential below −30 mV have excellent stability against flocculation or coalescence. The stable droplet size distribution during storage also reaffirms the absence of flocculation or coalescence.

### 3.4. Viscosity

The experimental data for all five emulsion systems was best fitted to a Newtonian model (R^2^ > 0.99), which indicated that all emulsions behaved as a Newtonian fluid. This observation was expected as a result of high water content, low protein concentration, and absence of polysaccharides and gums in the emulsions.

**Figure A1:**
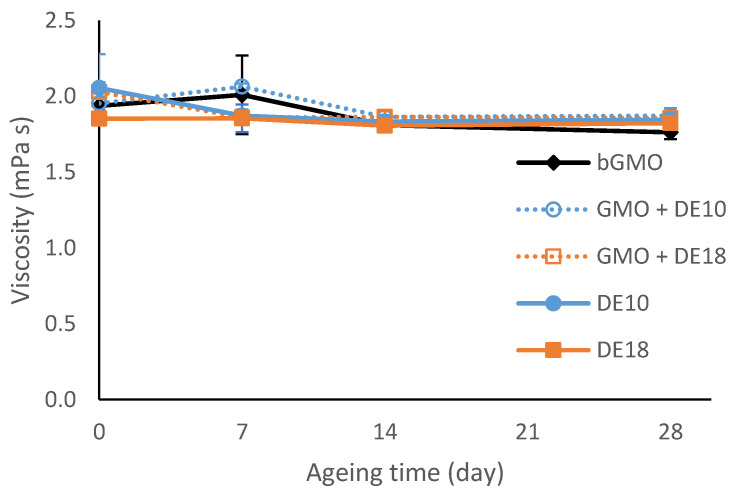
Viscosity of model emulsions formulated with different emulsifier systems at various storage times. Points represent average viscosity and the error bars are the standard deviation (*n* = 4; two batches × two replicates).

The fresh emulsion viscosity was not significantly affected by the emulsifier systems (*p* > 0.05) and were in the range of 1.8–2.1 mPa s (Figure A1). Hence, GMO did not affect the viscosity of emulsions and did not change the flow behavior, which agrees with previous findings [8]. All the emulsions also showed a small decline in viscosity after 28 days of storage to 1.7–1.9 mPa s (Figure A1).

### 3.5. pH

All fresh emulsions had similar pH in the range of 6.7–6.9, decreasing slightly (<0.2) over 28 days of storage with no superficial oil separation or protein aggregation. As expected, the inclusion of GMO at 0.2% did not affect the pH of the model emulsions because GMO is a non-ionic emulsifier that does not change the pH of the solution. The slight reduction in pH over the storage period could be due to precipitation of caseinate, which may also account for the small reduction in the ζ-potential.

### 3.6. Creaming Index

**Figure 3:**
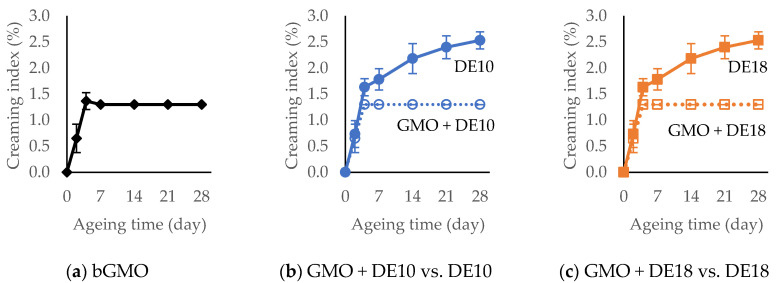
Creaming index of model emulsions formulated with different emulsifier systems at various storage times. Points represent average creaming index and the error bars represent standard deviation (*n* = 6; two batches × three replicates).

Figure 3 shows the creaming index of the model emulsions formulated with different emulsifier systems over 28 days of storage at 45 °C. A thin layer of cream started to develop in the emulsions with bulk GMO and instantized GMO powders (GMO + DE10 and GMO + DE18) after two days of storage at 45 °C and reached the maximum creaming index at 1.3% after seven days. The results also revealed that the sodium stearate from the instantized GMO powders did not affect creaming (i.e., compared to bulk GMO). In contrast, in the control emulsions with DE10 and DE18 the cream layer continued to increase after seven days, although at a slower rate and reached a creaming index of 2.4% at 28 days. This observation showed that emulsions with GMO had greater creaming stability than the controls, which agrees with previous findings [8] on creaming stability in protein-stabilized emulsions. The results showed that the DE value of maltodextrin had no effect on creaming stability. All emulsions had a similar protein composition and concentration, which indicated that the GMO component was the causative factor responsible for the improved creaming stability.

The formation of small droplets with a narrow size distribution in the emulsions was hypothesized to be due to the interaction between GMO and milk proteins, and was the key to form emulsions that were stable against creaming. According to Stokes’ Law, small droplets have a slower rate of creaming than larger ones and are less likely to separate due to gravitational forces [16]. Thus, a small average droplet size with a narrow droplet size distribution improves creaming stability for long-term storage [7,26,27,28].

### 3.7. Oxidative Stability of Model Emulsions Measured Using Volatile Analysis

The development of volatile compounds was monitored in all model emulsions with different emulsifier systems at various storage times using headspace SPME with GC-MS as a tool to evaluate changes in secondary oxidation products during storage. Volatile compounds that increased significantly (*p* < 0.05) during storage and had been reported as markers of lipid oxidation in the literature were reported in this study. Compounds with reduced or no change in concentrations during storage were not reported. Analysis of the volatile compounds detected 36 compounds (including two unknowns) that increased significantly in the model emulsions during storage in the model emulsions, from four chemical classes, namely aldehydes, furans, alcohols, and ketones. Hexanal was present with the highest abundance relative to other identified compounds.

**Figure 4:**
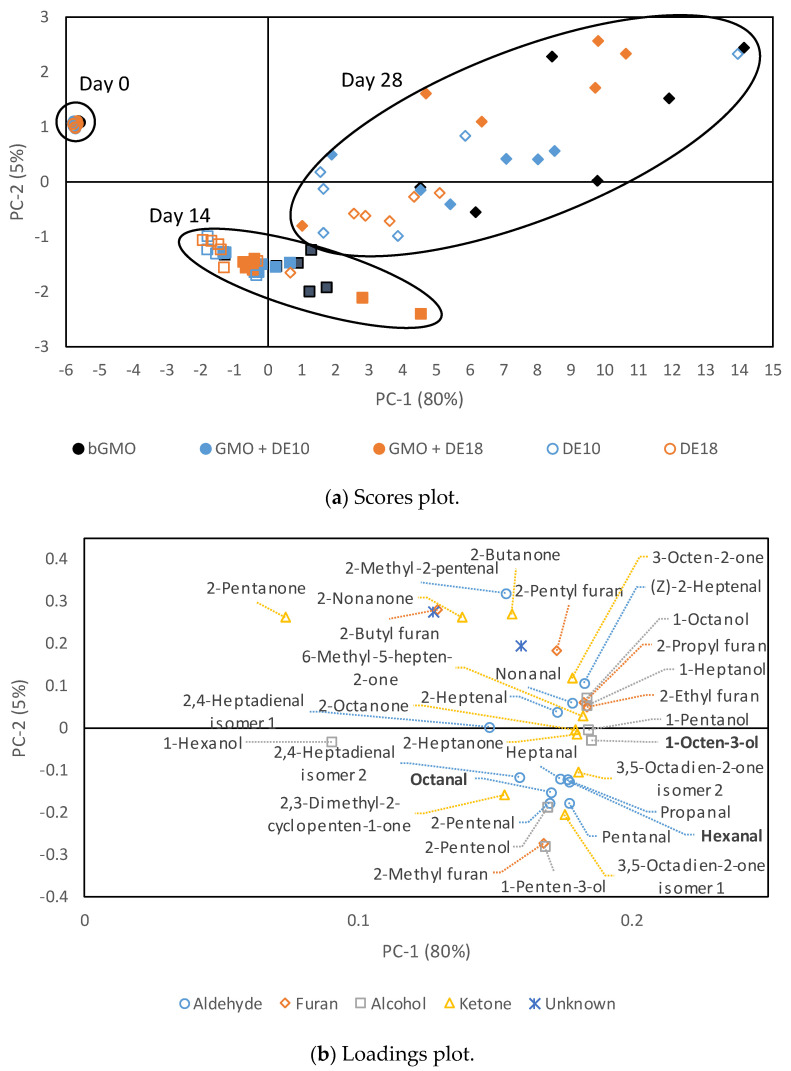
Principal component analysis (**a**) scores plot of sample formulations for model emulsions and (**b**) loadings plot of volatile compounds at various storage times. Each sample in the score plot has six replicate points (two batches × three replicates). Hexanal, octanal, and 1-octen-3-ol (bold text) were selected as oxidation markers.

Figure 4 shows the scores and loadings plot of principal component analysis (PCA) for the volatile analysis of the model emulsions at various storage times (Day 0, 14, 28). The first principal component (PC-1) explains most of the variation (80%) in the data and PC-2 explains 5% of the variation. Sample storage time (fresh vs. stored) was the main cause of data variation in the PCA model, and by using the first two principal components, the samples can be grouped into three clusters according to the storage time, namely Day 0, 14, and 28. Storage at 45 °C was the main cause of variation on PC-1 in the scores plot to differentiate fresh and stored emulsions. The discrimination on PC-1 was due to the abundance of alcohols (1-pentanol, 1-heptanol, 1-octanol, and 1 octen-3-ol), ketones (3-octen-2-one, 6-methyl-5-heptene-2-one, 2-heptanone, and 2 octanone), and furans (2-ethyl furan and 2-propyl furan), which were positively loaded on PC-1. PC-2 was able to distinguish the samples stored at 14 and 28 days by aldehydes, such as propanal, pentanal, hexanal, heptanal, octanal, 2-pentenal, and 2,4-heptadienal, which clustered together at the bottom of the loading plot that were associated with higher concentrations at Day 14.

The fresh control emulsions had eight volatile compounds that significantly increased during storage, namely pentanal, hexanal, heptanal, nonanal, 2,4-heptadienal isomer 2, 1-penten-3-ol, and two isomers of 3,5-octadien-2-one. In addition to the compounds that appeared in the controls, the fresh emulsions with bulk and instantized GMO had additional compounds, including 2-heptenal, 1-pentanol, 1-heptanol, 1-octanol, and 1-octen-3-ol. The number of compounds that significantly increased during storage increased to 35 compounds in the control emulsions after 28 days of storage, while the emulsions with GMO had 36 compounds. None of the furan compounds were detected in the fresh emulsions.

To investigate more closely the difference in the degree of oxidation between the different emulsions, three compounds, namely hexanal, octanal, and 1-octen-3-ol, were selected as oxidation markers. These compounds have been commonly used as lipid oxidation markers in past literature [10,15,29,30]. Hexanal and 1-octen-3-ol are formed by oxidation of linoleic acid, while octanal is derived from oleic acid [31,32]. Calibration curves for hexanal (R^2^ = 0.998), octanal (R^2^ = 0.996), and 1-octen-3-ol (R^2^ = 0.999) in the range of 0–900 µg/L were used for semi-quantification. Table 5 shows the concentration of the calibrated compounds at various storage times. The concentration of hexanal in the 28-day stored emulsions was outside of the calibration curve range and was determined by extrapolating the calibration curve beyond 900 µg/L with the assumption that the response continued to be linear.

All emulsions at Day 0 showed comparable low concentrations of hexanal and octanal, while 1- octen-3-ol was only detected in the emulsions with bulk GMO. This observation indicated that canola oil and GMO used to prepare the emulsions had a low degree of oxidation. The absence of 1-octen-3-ol in control emulsions (DE10 and DE18) indicated that this compound was present in the bulk GMO, while the non-detectable level in instantized GMO may be due to the evaporation during spray-drying. All three oxidation markers increased with the increasing storage time, which aligned with the PCA results, where compounds were positively loaded on PC-1 (Figure 4). The oxidation markers in the 28-day aged emulsions did not show any effect of the emulsifier system on oxidative stability. Table 6 shows additional 12 selected lipid oxidation compounds in the 28-day aged emulsions derived from oleic, linoleic, and linolenic acids [31] that had high loadings on PC-1. In bulk GMO, four compounds, namely propanal, 2,4-heptadienal isomer 2, 3 octen-2-one, and 3,5-octadien-2-one isomer 1, were significantly higher in concentration than in the controls (*p* < 0.05). All selected compounds, except propanal, indicated that there was no difference between instantized GMO samples and the controls. This observation was positive because it indicated that instantized GMO at a low concentration did not negatively affect the oxidative stability of protein-stabilized emulsions.

There was no influence of emulsion droplet size on lipid oxidation, which agrees with findings by Osborn and Akoh [33] and Dimakou et al. [34]. All emulsions also had very similar ζ-potentials, which indicated that the emulsions had similar protein composition in the aqueous phase and at the emulsion interface. Previous research [15] on the influence of protein composition on physical stability hypothesized that the formation of a compact multilayer interface comprised of sodium caseinate and whey proteins could improve oxidative stability in lipid emulsions. In this study, the emulsions with bulk and instantized GMO had the same oxidative stability as controls without GMO. The same protein composition at the interface indicated by the ζ-potential could have a bigger influence on oxidative stability compared to GMO at a low concentration and mask any effect by the GMO.

## 4. Conclusions

This study investigated the influence of instantized GMO powders on physical and chemical properties, creaming stability, and oxidative stability in protein-stabilized model emulsions. Instantized GMO formed emulsions with smaller mean droplet sizes and narrower size distributions than control emulsions (no GMO), which was similar to the emulsions with bulk GMO. The small droplet sizes with narrow distribution ranges resulted in greater stability against creaming. Maltodextrin in the emulsions did not show any effect on droplet size distribution or creaming stability. All fresh emulsions showed a similar volatile profile after microfluidization that changed significantly over time during storage. The volatile profile of the emulsions after 28 days of storage were not affected by GMO. These results reaffirmed that GMO plays a role in improving droplet size distribution that enhanced creaming stability in the emulsions. The smaller droplet size of the emulsions did not have any detrimental effect on oxidative stability. Instantized GMO powder retained all the functionality of bulk GMO and showed additional advantages such as stable emulsion properties after reconstitution, direct dispersion in aqueous formulation, ease of handling, and a longer shelf life compared to bulk GMO. This demonstrates the potential of this instantized GMO ingredient to function as secondary emulsifying and stabilizing agents that improve creaming stability of protein-stabilized emulsions during storage.

## Figures and Tables

**Table 1:** Formulation (based on % dry weight) and properties of instantized glycerol monooleate (GMO) powder.

Sample	GMO + DE10	GMO + DE18
**(a) Formulation of Instantized GMO Powders ^a^**		
**Core Materials**	**48.6%**	**48.6%**
(i) Glycerol monooleate	33.6%	33.6%
(ii) Canola oil	15.0%	15.0%
**Wall Materials**	**51.4%**	**51.4%**
(iii) Sodium caseinate	3.0%	3.0%
(iv) Sodium stearate	1.4%	1.4%
(v) Maltodextrin DE 10	47.0%	
(vi) Maltodextrin DE 18		47.0%
**(b) Properties of Instantized GMO Powders**		
Encapsulation efficiency (%) ^b^	93.3 ± 1.0	93.9 ± 1.2
Surface oil (%) ^c^	3.0 ± 0.4	3.1 ± 0.6
Moisture (%)	2.1 ± 0.6	2.1 ± 0.5
Dispersibility (%) ^d^	74.0 ± 0.8	87.3 ± 2.0
Initial droplet diameter (nm) ^e^	153.1 ± 2.7	153.9 ± 2.8
Reconstituted droplet diameter (nm) ^e^	267.1 ± 8.4	295.0 ± 11.8

^a^ DE, Dextrose equivalent. The ratio of GMO: Canola oil: NaCas was fixed at 33.6:15:3; ratio of core: wall at 0.95; and ratio of sodium caseinate: maltodextrin at 0.06 for both samples. Total solids were adjusted to 40% *w/w* solids during the emulsion preparation. ^b^ Encapsulation efficiency refers to the amount of oil-soluble ingredients encapsulated by the wall materials. ^c^ Surface oil content is the amount of extracted surface oil by hexane. ^d^ Dispersibility is the amount of reconstituted powder that can pass through a 250 μm test sieve. ^e^ Droplet diameter is the average droplet diameter measured using dynamic light scattering.

**Table 2:** Formulation table for preparing model emulsion samples.

Sample	bGMO	GMO + DE10	GMO + DE18	DE10	DE18
**(a) Oil Phase**					
Canola oil	4.0%	4.0%	4.0%	4.0%	4.0%
GMO	0.2%				
**(b) Water Phase**					
Caster sugar	6.0%	6.0%	6.0%	6.0%	6.0%
Sodium caseinate	0.80%	0.80%	0.80%	0.80%	0.80%
WPC	0.20%	0.20%	0.20%	0.20%	0.20%
Sodium azide	0.02%	0.02%	0.02%	0.02%	0.02%
Maltodextrin DE 10				0.28%	
Maltodextrin DE 18					0.28%
GMO + DE10 powder		0.60%			
GMO + DE18 powder			0.60%		
Deionized water	88.8%	88.4%	88.4%	88.7%	88.7%

GMO, Glycerol monooleate; bGMO, bulk GMO; WPC, Whey protein concentrate; DE, Dextrose equivalent.

**Table 3:** Droplet size and polydispersity index of model emulsions formulated with different emulsifier systems at various storage times.

Sample	Day 0	Day 7	Day 14	Day 28
**(a) Droplet Size (nm)**				
bGMO	186.0 ± 5.6 ab	183.2 ± 5.2 a	182.3 ± 4.0 a	184.1 ± 3.1 a
GMO + DE10	178.1 ± 1.5 a	175.7 ± 2.3 a	175.1 ± 3.2 a	177.9 ± 2.3 a
GMO + DE18	175.8 ± 0.8 a	174.1 ± 0.6 a	172.3 ± 1.0 a	174.0 ± 0.9 a
DE10	198.4 ± 8.5 c	199.8 ± 10.5 b	196.4 ± 9.2 b	199.5 ± 9.6 b
DE18	196.9 ± 6.1 bc	196.1 ± 5.0 b	194.7 ± 4.4 b	198.4 ± 3.5 b
F-value	15.2	16.3	18.7	22.7
*p*-value	<0.001	<0.001	<0.001	<0.001
**(b) Polydispersity index**				
bGMO	0.162 ± 0.022 ab	0.170 ± 0.012 a	0.177 ± 0.009 a	0.177 ± 0.019 a
GMO + DE10	0.163 ± 0.019 ab	0.162 ± 0.015 a	0.162 ± 0.008 a	0.165 ± 0.010 a
GMO + DE18	0.158 ± 0.014 a	0.158 ± 0.006 a	0.161 ± 0.009 a	0.160 ± 0.006 a
DE10	0.201 ± 0.022 b	0.204 ± 0.022 b	0.202 ± 0.021 b	0.202 ± 0.018 b
DE18	0.194 ± 0.015 ab	0.209 ± 0.009 b	0.198 ± 0.011 b	0.200 ± 0.018 b
F-value	4.6	11.5	10.0	6.6
*p*-value	<0.05	<0.001	<0.001	<0.01

Values represent average ± standard deviation of four measurements (two batches × two replicates). Different letters in the same column indicate statistically significant differences (*p* < 0.05) by Tukey’s post hoc multiple comparison test.

**Table 4:** ζ-potential of model emulsions formulated with different emulsifier systems at various storage times.

Sample	Day 0	Day 7	Day 14	Day 28
bGMO	−51.6 ± 1.4 a	−47.8 ± 0.5 c	−47.7 ± 0.4 a	−46.9 ± 1.1 b
GMO + DE10	−51.9 ± 0.2 a	−49.1 ± 0.3 b	−49.0 ± 0.5 b	−47.6 ± 0.2 ab
GMO + DE18	−52.1 ± 0.4 a	−49.0 ± 0.3 b	−49.2 ± 0.5 b	−47.7 ± 0.1 ab
DE10	−53.0 ± 0.3 a	−49.8 ± 0.5 ab	−49.9 ± 0.7 b	−48.1 ± 0.9 ab
DE18	−52.9 ± 0.8 a	−50.3 ± 0.0 a	−50.0 ± 0.8 b	−49.3 ± 1.1 a
F-value	2.7	16.9	10.0	4.8
*p*-value	0.072	<0.001	<0.001	<0.05

Values represent average ± standard deviation of four measurements (two batches × two replicates). Different letters in the same column indicate statistically significant differences (*p* < 0.05) by Tukey’s post hoc multiple comparison test.

**Table 5:** Lipid oxidation markers for fresh and 28-day aged (45 °C) emulsions as a function of the emulsifier system.

Retention Index	Volatile Compound	Day	Concentration (µg/L) ^a^					Source of Lipid Oxidation [31]
			bGMO	GMO + DE10	GMO + DE18	DE10	DE18	
1082	Hexanal	0	25.0 ± 8.4 a	17.7 ± 7.1 a	21.9 ± 6.1 a	16.2 ± 8.8 a	18.7 ± 6.0 a	n-6 linoleic acid
		14	567.8 ± 118.8 bc	490.7 ± 102.7 abc	670.1 ± 294.3 c	294.8 ± 121.1 a	361.9 ± 20.8 ab	
		28	945.0 ± 364.6 a	819.7 ± 309.6 a	902.4 ± 266.7 a	739.5 ± 262.5 a	726.8 ± 143.8 a	
1292	Octanal	0	n.d.	n.d.	n.d.	n.d.	n.d.	n-9 oleic acid
		14	180.8 ± 52.4 a	141.0 ± 18.6 a	226.0 ± 145.4 a	145.2 ± 24.7 a	166.2 ± 82.4 a	
		28	380.3 ± 111.3 a	292.0 ± 68.5 a	297.7 ± 66.5 a	232.1 ± 77.6 a	273.4 ± 68.6 a	
1441	1-Octen-3-ol	0	2.1 ± 0.1	n.d.	n.d.	n.d.	n.d.	n-6 linoleic acid
		14	28.7 ± 4.2 a	26.1 ± 2.9 a	29.5 ± 8.1 a	19.4 ± 1.5 a	20.0 ± 1.9 a	
		28	60.3 ± 15.8 a	49.8 ± 8.7 a	54.7 ± 14.9 a	43.4 ± 18.4 a	38.9 ± 5.6 a	

^a^ Values are average concentration (µg/L) ± standard deviation of six measurements (two batches × three replicates). Different letters in the same row indicate statistically significant differences (*p* < 0.05) by Tukey’s post hoc multiple comparison test. n.d.: Not detected.

**Table 6:** Lipid oxidation compounds in 28-day aged (45 °C) emulsions as a function of the emulsifier system.

Retention Index	Compound	TIC Peak Area (Million au) ^a^				
		bGMO	GMO + DE10	GMO + DE18	DE10	DE18
794	Propanal	33.11 ± 6.62 c	28.12 ± 6.23 bc	30.96 ± 7.61 c	18.28 ± 4.98 ab	16.22 ± 2.54 a
956	2-Ethyl furan	171.70 ± 41.12 a	129.93 ± 34.55 a	137.76 ± 41.87 a	132.89 ± 79.60 a	101.67 ± 22.18 a
982	Pentanal	56.61 ± 13.02 a	48.06 ± 13.51 a	43.86 ± 11.09 a	46.29 ± 20.04 a	42.55 ± 7.87 a
1232	2-Pentyl furan	85.45 ± 31.15 a	55.89 ± 17.00 a	60.65 ± 16.57 a	60.20 ± 58.76 a	38.99 ± 7.59 a
1278	(Z)-2-Heptenal	14.51 ± 4.85 a	11.11 ± 2.71 a	12.76 ± 4.90 a	9.55 ± 4.06 a	8.99 ± 1.85 a
1397	Nonanal	38.51 ± 12.78 a	30.27 ± 7.12 a	31.51 ± 9.50 a	25.94 ± 17.65 a	21.06 ± 7.23 a
1414	3-Octen-2-one	52.50 ± 21.47 b	38.44 ± 8.94 ab	43.51 ± 17.97 ab	23.13 ± 9.97 a	21.48 ± 5.39 a
1448	1-Heptanol	55.12 ± 17.99 a	38.75 ± 7.59 a	44.75 ± 15.39 a	34.80 ± 21.37 a	30.01 ± 8.11 a
1505	2,4-Heptadienal isomer 2	14.27 ± 5.21 b	9.44 ± 1.57 ab	10.36 ± 3.00 ab	6.73 ± 3.59 a	5.25 ± 0.80 a
1527	3,5-Octadien-2-one isomer 1	94.63 ± 22.08 b	77.37 ± 10.67 ab	85.60 ± 19.21 ab	66.08 ± 14.70 a	59.94 ± 5.82 a
1551	1-Octanol	29.96 ± 10.32 a	21.77 ± 4.74 a	25.05 ± 8.87 a	19.16 ± 12.92 a	16.78 ± 4.37 a
1581	3,5-Octadien-2-one isomer 2	338.93 ± 54.61 a	294.74 ± 32.67 a	315.95 ± 59.86 a	280.03 ± 41.43 a	264.48 ± 25.33 a

^a^ Values represent average peak area ± standard deviation of six measurements (two batches × three replicates). Different letters in the same row indicate statistically significant differences (*p* < 0.05) by Tukey’s post hoc multiple comparison test.
